# Managing expectations with psychedelic microdosing

**DOI:** 10.1038/s44184-023-00044-9

**Published:** 2023-11-08

**Authors:** Omer A. Syed, Benjamin Tsang

**Affiliations:** 1 Institute of Medical Science, University of Toronto, Toronto, Canada; 2 Department of Cell & Systems Biology, University of Toronto, Toronto, Canada

**Keywords:** Outcomes research, Human behaviour

## Abstract

Microdosing psychedelics is a growing practice among recreational users, claimed to improve several aspects of mental health, with little supporting empirical research. In this comment, we highlight the potential role of expectations and confirmation bias underlying therapeutic effects of microdosing, and suggest future avenues of research to address this concern.

## Introduction

The recent years have seen an unprecedented surge of psychedelic research. Studies with psilocybin (colloquially known as magic mushrooms), in particular, have shown promise in ameliorating symptoms of treatment-refractory depression, anxiety, and substance use disorder, among others^1,2^. Microdosing, the practice of regularly taking roughly a tenth of full psychedelic doses, is becoming increasingly popular^3^. These sub-perceptual doses cause no psychedelic effects, yet anecdotal reports suggest they carry substantial improvements to mental wellbeing. Popularized in-part by Silicon Valley developers and entrepreneurs, microdosing psilocybin and LSD is widely suggested to boost creativity, attention, sociability, while treating low mood and anxiety. Additionally, recent years have seen microdosing permeating the mainstream psychedelic scene. Several recent self-report survey studies demonstrate the overwhelming positive effects of microdosing on users’ mental health^3,4^.

While there is also emerging literature on the benefits of psychedelics in psychiatry, the vast majority of clinical studies use large psychedelic doses^5^. Consequently, there is little clinical data on microdosing to test the veracity of the claims and trends depicted in self-report surveys. In this comment, we discuss how positive expectations towards microdosing may be responsible for the reported benefits, the downsides of the expectancy bias, and outline future research to validate the true pharmacological effects of microdoses.

## Effects of expectations and settings on positive outcomes

Consider the example of Barry, whose mental health has taken a hit since the pandemic. He has lost interest in a lot of his hobbies, finds it harder to focus at work, and low mood persists much longer than before. His friends recommend microdosing psilocybin, and their enthusiastic endorsements pique his interest, spurring him to into it online. A cursory search finds TED Talks that discuss the efficacy of psilocybin in treating a range of psychiatric conditions, and popular influencers extolling the benefits of microdosing on their mental health. He also reads up on the concept of ‘Set and Setting’, which highlights the importance of being in a positive mindset and a supportive and calm environment while taking psychedelics^6^.

Intrigued and hopeful, Barry decides to try microdosing. Before his first microdose, he writes out his intentions behind using psychedelics in his journal, thinks about what he expects to get out from this experience, plays his favorite calming music, and makes himself comfortable in his room. After a few weeks of following a microdosing regimen, he feels a lot more connected to his work and his peers, and the negative thoughts have blissfully dampened. Microdosing surely did its work. But did it really?

It is hard to be sure that the psilocybin microdoses’ pharmacological properties are solely (or significantly) responsible for Barry’s improved mental health. It is important to note how Barry, perhaps alongside many others that found benefits, began microdosing with preconceived positive expectations associated with the practice. Either from word of mouth or the disproportionate hype around psychedelics online, many people start microdosing with the expectation of positive outcomes. We should also consider the potential influence of Set and Setting itself. Although the primary reason for having a positive mindset and a safe space while taking psychedelics is to avoid bad trips, taking time out of your day to ensure a positive mindset and being in a calming space may be therapeutic by itself. Indeed, individuals simply self-isolating and meditating in stress-free natural settings have reported meaningful experiences, while devoid of any psychedelic use^7^. With sparse literature on controlled studies with microdosing, we have little understanding on the extent of therapeutic responses that are owed to the pharmacological effects of psychedelic microdoses. In fact, it is entirely plausible to think that extra-pharmacological factors such as positive expectations and surrounding yourself in a calming environment may also be largely responsible for the observed improvements in mental health.

The placebo and expectancy bias is also widespread in psychedelic research. Many participants in a study by Olson and colleagues^8^, for instance, reported experiencing altered visual effects when given a cellulose placebo pill under the pretense of it being psychedelic mushrooms. Several other studies have signaled towards expectancy effects in microdosing as well^9,10^. Importantly, however, the expectancy effect is common and not at all unique to psychedelics; it is even true for coffee. A study similar to the aforementioned, where participants received a decaffeinated drink disguised as caffeine experienced a boost in their mood^11^. The key difference is that the pharmacodynamics of caffeine are well-established, and our understanding of its effects on our neurophysiology is deep, whereas this is not yet the case for psychedelic microdoses.

It is important to make the distinction between the effects caused by the expectancy bias and the drug-induced pharmacological effects on the body. This distinction, however, may be of more interest to researchers and policy makers, than the average user. In Barry’s case, for instance, he is likely to be unconcerned with the intricacies of the underlying neurophysiological processes, or lack thereof, that contributed to his improved mood. He’s going to continue his regimen as long as he experiences these improvements. Does it matter if these improvements in mental health are a consequence of environmental conditions and expectations masked as meaningful pharmacological effects? We believe it does.

## The implications of the expectancy effect

Consider the idea that microdosing has no meaningful pharmacological effects. Although many people may experience significant improvements in their mental health from self-medicating psychedelic microdoses, such benefits driven by positive expectations would be merely a temporary fix; a Band-Aid on a bullet wound. These short-lived perceived benefits may delay users from receiving proven effective treatments, possibly worsening their untreated symptoms in the long run. Another concern with an expectancy effect is that, when on a regimen for a while and it becomes part of a routine, the strength of the expectations may erode over time, and the declining improvements shortly follow. When a seemingly ‘effective’ microdosing practice stops working, people may increase the frequency, believing it to be correlated with improvements. There are several outstanding safety concerns with this practice; not allowing an adequate and regular wash-out period, and an over-activation of the serotonergic system which is linked to potential cardiac complications^12^. Although the extent of these risks are not fully substantiated for most classical psychedelics, it is a current area of discussion.

So far, we have presented the idea of microdosing and psychedelic use associated with improving wellbeing. For any drug, however, there will always be non-responders, regardless of the level of expectations or placebo attached to it; psychedelics are no exception. The building of excessive positive expectations, therefore, may do more harm than good. Consider individuals with severe treatment-refractory depression that have found no relief from a range of traditionally effective modalities. When they come across social media and the news endorsing psychedelic-therapies as revolutionary psychiatric treatments, they may either sign-up for clinical trials or try self-medicating on their own time while expecting benefits. Unfortunately, those who fail to notice any significant improvements in their symptoms following their engagement in such psychedelic therapies could be vulnerable to a worsened emotional state. A similar phenomenon potentially occurred in a recent trial of psilocybin-assisted psychotherapy for depression^13^, which found reports of suicidal behavior among three participants following the study; all being non-responders^14^. Although this trial did not administer microdoses, the idea still holds true: just as positive expectations may underlie positive outcomes, they may also easily cause the reverse.

## Navigating policy decisions with hype and expectations

Although the FDA has not re-classified psychedelic substances since the change in 1970, there are trends for new public policies in the area. The promising results of recent clinical findings may be why Australia and states like Oregon are changing policies to legalize medical use of psychedelics. In other places psychedelics may also soon be on the path of legalization or decriminalization. While there is a strong case opposing the schedule I classification of psychedelics^15^, there are still many unknowns, and acknowledging these gaps in knowledge are incredibly important when evolving health policies and law in some places seem to be outpacing the research.

The changes in policies are undoubtedly influenced, at least in part, by the increasing public interest surrounding psychedelics. As new clinical studies indicate psychedelics as promising in treating various psychiatric conditions, the extensive media coverage and hype on the topic portray psychedelics as a panacea, although the research field largely remains in its infancy. While it is true that clinical trials have consistently signaled towards psychedelics to elicit positive outcomes in mental health, the hype surrounding psychedelics is likely to bias those applying to these studies. Open-label studies with participants that apply and enroll with preconceived positive expectations are surely to influence the results. Moreover, these positive findings may fuel recreational use as well, especially sub-hallucinogenic microdosing, which is considered a novice’s entry into psychedelic use.

Meanwhile legislators and psychedelic advocates may point towards the positive findings of clinical trials and the increasing trends of beneficial recreational use as justification for legalizing or decriminalizing psychedelic use. Although the aforementioned is certainly welcomed to an extent, we emphasize that the opposite can easily occur whereby widespread illegal recreational use drives stigmatization and potential health risks, thereby leading to a hard-lined response from governmental agencies impeding future developments in psychedelic research. As mentioned earlier, though public policy on the area is evolving, we hope it is not fueled by inflated positive findings and outpace objective research in the area.

## Future outlook

In this comment, we echo the concerns surrounding expectancy effects prevailing in the psychedelic microdosing scene. Many users claim improved mental health and well-being with microdosing, but this may be largely driven by their preconceived positive expectations. Positive expectations, however, are also likely to be prevalent in clinical studies which may inadvertently skew the results of studies with larger doses. Unfortunately, there is little we can do to mask the positive media coverage and hype surrounding psychedelics, although we argue that proper dissemination of data and education to reporters are a step in the right direction. While the psychedelic field is undoubtedly an exciting and promising frontier, perhaps a more balanced and rational view should be taken in public forums. Nevertheless, clinical studies may benefit from administering validated scales such as the Expectations for Treatment Scale^16^, or Credibility/Expectancy Questionnaire^17^, which provide insights into the degree of preconceived expectations within participants while entering the study. The results of these questionnaires can be analyzed along with treatment outcomes to delineate the extent of influence that expectations hold over therapeutic responses.

As researchers with a background in animal research, our instinct is to turn towards animal models for insights. Free of being swayed by popular culture, expectations or placebos, animal models–rats, mice, and zebrafish–can all uncover the neurophysiological effects of microdosing on neurological and behavioral endpoints. Although previously employed for this topic^18^, the literature is still too scarce to generate any meaningful conclusions. In the coming years, we stress the importance of distinguishing the improvement in mental health as a consequence of positive expectations and the true underlying physiological changes elicited by microdosing psychedelics.
